# Deep Learning-Assisted Measurements of Photoreceptor Ellipsoid Zone Area and Outer Segment Volume as Biomarkers for Retinitis Pigmentosa

**DOI:** 10.3390/bioengineering10121394

**Published:** 2023-12-06

**Authors:** Yi-Zhong Wang, Katherine Juroch, David Geoffrey Birch

**Affiliations:** 1 Retina Foundation of the Southwest, 9600 North Central Expressway, Suite 200, Dallas, TX 75231, USA; kjuroch@retinafoundation.org (K.J.); dbirch@retinafoundation.org (D.G.B.); 2 Department of Ophthalmology, University of Texas Southwestern Medical Center, 5323 Harry Hines Blvd, Dallas, TX 75390, USA

**Keywords:** deep learning, retinitis pigmentosa, photoreceptor ellipsoid zone area, photoreceptor outer segment volume, biomarkers

## Abstract

The manual segmentation of retinal layers from OCT scan images is time-consuming and costly. The deep learning approach has potential for the automatic delineation of retinal layers to significantly reduce the burden of human graders. In this study, we compared deep learning model (DLM) segmentation with manual correction (DLM-MC) to conventional manual grading (MG) for the measurements of the photoreceptor ellipsoid zone (EZ) area and outer segment (OS) volume in retinitis pigmentosa (RP) to assess whether DLM-MC can be a new gold standard for retinal layer segmentation and for the measurement of retinal layer metrics. Ninety-six high-speed 9 mm 31-line volume scans obtained from 48 patients with RPGR-associated XLRP were selected based on the following criteria: the presence of an EZ band within the scan limit and a detectable EZ in at least three B-scans in a volume scan. All the B-scan images in each volume scan were manually segmented for the EZ and proximal retinal pigment epithelium (pRPE) by two experienced human graders to serve as the ground truth for comparison. The test volume scans were also segmented by a DLM and then manually corrected for EZ and pRPE by the same two graders to obtain DLM-MC segmentation. The EZ area and OS volume were determined by interpolating the discrete two-dimensional B-scan EZ-pRPE layer over the scan area. Dice similarity, Bland–Altman analysis, correlation, and linear regression analyses were conducted to assess the agreement between DLM-MC and MG for the EZ area and OS volume measurements. For the EZ area, the overall mean dice score (SD) between DLM-MC and MG was 0.8524 (0.0821), which was comparable to 0.8417 (0.1111) between two MGs. For the EZ area > 1 mm^2^, the average dice score increased to 0.8799 (0.0614). When comparing DLM-MC to MG, the Bland–Altman plots revealed a mean difference (SE) of 0.0132 (0.0953) mm^2^ and a coefficient of repeatability (CoR) of 1.8303 mm^2^ for the EZ area and a mean difference (SE) of 0.0080 (0.0020) mm^3^ and a CoR of 0.0381 mm^3^ for the OS volume. The correlation coefficients (95% CI) were 0.9928 (0.9892–0.9952) and 0.9938 (0.9906–0.9958) for the EZ area and OS volume, respectively. The linear regression slopes (95% CI) were 0.9598 (0.9399–0.9797) and 1.0104 (0.9909–1.0298), respectively. The results from this study suggest that the manual correction of deep learning model segmentation can generate EZ area and OS volume measurements in excellent agreement with those of conventional manual grading in RP. Because DLM-MC is more efficient for retinal layer segmentation from OCT scan images, it has the potential to reduce the burden of human graders in obtaining quantitative measurements of biomarkers for assessing disease progression and treatment outcomes in RP.

## 1. Introduction

Retinitis pigmentosa (RP) is one of the leading causes of irreversible vision loss among various types of inherited retinal degeneration. Retinal scan images obtained using optical coherence tomography (OCT) have shown that the structural changes in RP mostly occur in the outer retina [1,2,3]. One of the hallmarks of structural change in RP is the shrinking of the photoreceptor ellipsoid zone (EZ), or the junction of the photoreceptor inner and outer segments, with the progression of the disease. The change in EZ band width with disease progression is evident in cross-sectional OCT scan images (B-scans) when the EZ transition zone is within the scan limit [4]. Previous studies have suggested using EZ width [4] and EZ area [5] as biomarkers to evaluate disease progression and to predict vision loss in RP. A recent study showed that patients with X-linked RP (XLRP) exhibited a shortening of the photoreceptor outer segment (OS) in the early stage of the disease [6]. Hence, quantitative measurements of three-dimensional (3D) OS metrics, including OS length, OS (or EZ) area, and OS volume, may provide a more comprehensive set of biomarkers for assessing disease progression. However, one of the challenges involved in obtaining reliable 3D OS metrics measurements is the accurate delineation of the OS boundaries, including both the EZ band and the proximal retinal pigment epithelium (pRPE) on all B-scan images in a volume scan. Current conventional automatic OCT segmentation algorithms often incorrectly identify the EZ transition zone and thus require manual segmentation by human graders for accurate identification of the OS, which is time-consuming and costly [7], especially for a high-density volume scan which may contain more than one hundred B-scans.

Recent advances in machine learning have provided new tools for clinical applications in ophthalmology, especially in retinal diseases [8,9,10,11,12,13,14,15]. One such application is the automated layer segmentation in OCT scan images [16,17,18,19,20,21,22,23,24], in particular automatic measurements of EZ width or area from the retinal layer segmentation of OCT scan images in patients with inherited retinal degeneration [7,25,26,27,28]. For instance, Camino et al. implemented a method based on a convolutional neural network (CNN) for the segmentation of a preserved EZ area on en face OCT in choroideremia and RP and achieved 90% accuracy [25]. Loo et al. evaluated a deep learning-based algorithm originally developed for macular telangiectasia [29] for the segmentation of the EZ area in USH2A-related RP [26], and they showed that the deep learning algorithm performed well in segmenting the EZ area, with a dice similarity score (SD) of 0.79 (0.27). Wang et al. developed a machine learning method based on random forests for the automatic detection of continuous areas of a preserved EZ in choroideremia [28]. We also developed a hybrid deep learning model (DLM) that combined two CNNs of different structures, a U-Net for fast semantic segmentation and a sliding-window CNN for refinement, for the segmentation of five retinal layer boundaries, including both the EZ and pRPE, in OCT B-scan images, and demonstrated that the model performed well in EZ width measurement when compared to human graders [27,30]. While new machine learning algorithms have shown promise for the automatic measurement of EZ width and/or area, OS volume was not determined in these studies.

One of the features of our DLM is that, from the EZ and pRPE segmentation in each B-scan of a volume scan, a 3D OS formation can be reconstructed to obtain the measurements of OS thickness, EZ area, and OS volume. Using this approach, we demonstrated that the EZ area measurements obtained with our DLM were in good agreement with those by a reading center [7]. In a more recent study, where the longitudinal changes in OS metrics were examined in XLRP, a total of 342 OCT volume scans were segmented automatically by our model, then manually checked and corrected by human graders for the EZ and RPE to obtain OS metrics measurements, demonstrating for the first time that with the assistance of deep machine learning, it is possible to measure OS volume in a large dataset and significantly reduce the time needed for manual segmentation [31]. Hence, DLM segmentation with manual correction (DLM-MC) could potentially be a new gold standard for OS metrics measurements to ease the burden of human graders. The question is “how well does DLM-MC perform when compared to the conventional manual grading (MG) by human graders?” It was shown in our previous study that increasing the size of the training dataset, especially by adding more cases with the EZ band extended towards and beyond the edge of the macula, can improve the model’s performance [7]. The question is “can we improve DLM performance further with additional training data so that eventually the performance of DLM alone without manual correction can be closer enough to that of MG?” Furthermore, our studies so far employed a single image patch size of 256 × 32 for the U-Net model training. It is unknown what the effect is of different image patch sizes on the performance of trained models. Hence, in this study, we compared the performance of DLM-MC to that of MG for the measurement of EZ area and OS volume. We also assessed the impact of increasing the training dataset sizes as well as changing the training image patch sizes on the performance of the DLM only for the measurement of EZ area and OS volume.

## 2. Materials and Methods

### 2.1. Test Dataset—OCT Volume Scans and Manual Segmentation

**Figure 1:**
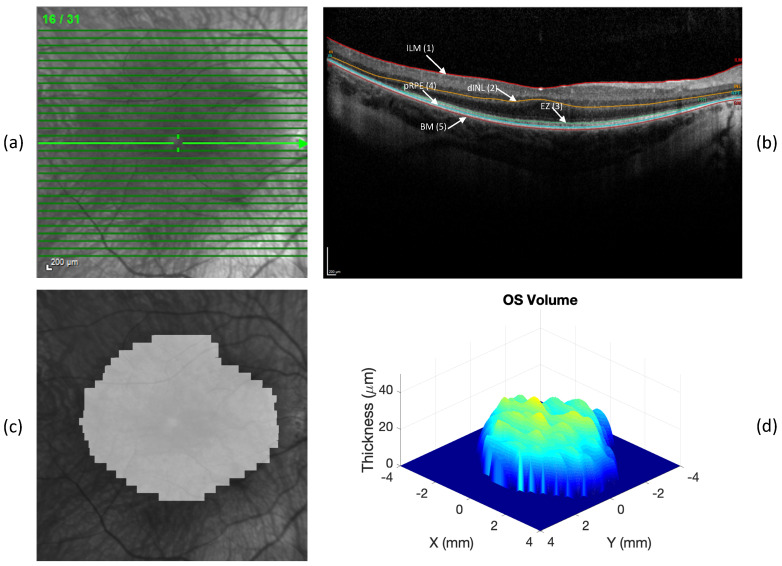
An example of OCT volume scan obtained with Spectralis SD-OCT device (Heidelberg Engineering, Heidelberg, Germany). (**a**) Infrared fundus image with 31 lines of B-scan; (**b**) mid-line B-scan from (**a**), showing five retinal layer boundaries labelled: inner limiting membrane (ILM), distal inner nuclear layer (dINL), ellipsoid zone (EZ), proximal retinal pigment epithelium (pRPE), and Bruch’s membrane (BM); (**c**) EZ area overlapped with infrared fundus image; (**d**) OS volume map. Note that the *z*-axis in volume map is in micrometer, different from the *x*-axis and *y*-axis in mm.

Ninety-six high-speed 9 mm 31-line volume scans obtained from 48 patients with retinitis pigmentosa GTPase regulator (RPGR)-associated XLRP (one volume scan per eye) were selected based on the following criteria: the presence of an EZ band transition zone within the scan limit and a detectable EZ in at least 3 B-scans in a volume scan. All the volume scans were obtained using a Heidelberg Spectralis HRA-OCT (Heidelberg Engineering, Heidelberg, Germany). Figure 1 shows an example of a volume scan obtained from a patient with RP. These volume scans were used as a testing dataset for comparing the performance of DLM-MC, as well as the performance of the DLM only, with that of MG in the measurements of EZ area and OS volume. A subset of these patients (n = 39) participated in a previous study that examined the effect of docosahexaenoic acid (DHA) on the disease progression [32].

All the B-scan images in each volume scan were manually segmented for the EZ and proximal (apical) retinal pigment epithelium (pRPE) by two experienced human graders. The manual segmentation was conducted using Heidelberg Eye Explorer software (version 1.9.10.0). Any prior segmentation of retinal layers in all the volume scans was removed before performing manual grading for this study. The manual tracing of EZ and pRPE in a B-scan image followed the conventions described in a previous study [4]. The manual segmentation served as the ground truth for the comparison with other segmentation methods, including DLM-MC and the DLM only.

### 2.2. Deep Learning Model and Training Datasets

The deep learning model (DLM) employed in this study is the hybrid model we developed [27]; it consists of a U-Net CNN model [33] for the initial, fast semantic segmentation and a sliding-window (SW) CNN model [34] for refinement through the correction of the potential segmentation errors made by the U-Net. Our model was designed to identify five retinal boundaries: the inner limiting membrane (ILM), the distal inner nuclear layer (dINL), the EZ, the pRPE, and Bruch’s membrane (BM). The details of the model’s structures, implementation, and training and testing were reported previously [27,30], and a summary of the model was described in a more recent publication [7]. All the models were implemented in MATLAB (MathWorks, Natick, MA, USA) and trained on a Mac Pro desktop computer (Apple, Inc., Cupertino, CA, USA, 2019, 3.2 GHz 16-Core Intel Xeon W with 192 GB 2933 MHz DDR4 RAM).

In this study, four datasets were used for the model training and validation: (1) the original RP240 dataset [30]; (2) the RP340 dataset, as reported previously [7], which included the RP240 dataset with an additional 200 mid-line B-scans containing an increased number of cases with an extended EZ band obtained from 100 patients with RP; (3) the RP480 dataset, which was generated by adding 280 mid-line B-scans from 140 patients with RP to RP340 to double the size of the original dataset; (4) the RP140 dataset, which consisted of the above-mentioned 280 mid-line B-scans to give a size range for the training datasets. Only two B-scans, one per eye, were obtained from a patient in each dataset. All the mid-line B-scans, which were composed of a mix of high-speed (768 A-scans) and high-resolution (1536 A-scans) B-scans with an automatic real-time tracking (ART) setting of 100, were obtained using the Heidelberg Spectralis SD-OCT. There was no overlap of patients between the training datasets and the testing dataset described in Section 2.1.

For the U-Net, three patch sizes were used for the training images: 256 × 32, 256 × 64, and 256 × 128 pixels. The methods used for the extraction of the image patches and for data augmentation were reported previously [27].

For the SW model, the training data were tiny image patches of 33 × 33 pixels extracted from the B-scan images. The class of each patch was defined by the class of its center pixel. The patches generated for training were centered at the pixels on 5 boundary lines or centered on the background pixels [30]. The patches centered on the ILM, dINL, EZ, pRPE, or BM boundary lines were labelled as 1, 2, 3, 4, or 5, respectively. Any patches not centered on these five boundary lines were labeled as 0.

Table 1 lists the four training datasets with the details, including patient composition, the number of B-scans in each scan type, the patch image size for the U-Net, and the number of image patches for training and validation.

### 2.3. Deep Learning Model Training and Validation

The details of the model training and validation were reported previously [7,27,30]. All the labeled image patches were randomly divided into a training set (80%) and a validation set (20%). To accelerate the CNN training and reduce the sensitivity to network initialization [35], a batch normalization layer was inserted between the convolutional layers and the rectified linear unit (ReLU) layers for the SW model training and between the convolutional layers and the ReLU layers in the encoding subnetwork for the U-Net training. The U-Net and the SW model as a pair were trained on all four datasets. The U-Net training also included three different patch sizes for each dataset. The trained models were named after the names of the corresponding training datasets.

To account for the potential impact of the randomization of the initial filter weights and stochastic learning algorithms of the model training on the OS metrics measurements, all the models were trained three times on the same datasets [7].

### 2.4. Segmentation of Test Volume Scans by DLM-MC and DLM Only

For DLM-MC, all the B-scan images in each volume scan in the test dataset were first segmented automatically for five retinal boundary lines (ILM, dINL, EZ, pRPE, and BM) by the RP340 model; then, these B-scans were checked and manually corrected by the same two graders for potential errors made by the RP340 model for the boundary lines of the EZ and pRPE. The manual correction to the automatic segmentation of the DLM was performed using the software of the Manual Segmentation Utility created by the Hood Visual Science Laboratory (HVSL) [36].

For the DLM only, all the B-scan images in each volume scan in the test dataset were segmented automatically for the same five retinal boundary lines by the RP140, RP240, RP340, and RP480 models, respectively. As three image patch sizes were used for the U-Net training and each model was trained three times to account for the randomization of the initial weights of the model, each B-scan image was segmented a total of 36 times (4 × 3 × 3) by the various models.

### 2.5. Photoreceptor Outer Segment (OS) Metrics Measurements

The methods used to obtain the 3D OS metrics measurements (OS thickness, EZ area, and OS volume) were the same as previously reported [31]. An example of a 3D OS map as well as the EZ area can be found in Figure 1. The OS metrics measurements were then obtained from the reconstructed 3D OS maps. Specifically, the mean OS thickness was the average of all the non-zero single pixel OS thicknesses; the EZ area was estimated by multiplying the area of a single grid pixel by the number of pixels with a measurable OS; and the OS volume was the sum of the products of the OS thickness at each pixel and the pixel area. In this study, only the EZ area and OS volume measurements were evaluated since our recent study [31] suggested that average OS length alone may not be a good candidate as a biomarker due to the current limit of the axial resolution of OCT, which could result in a larger OS length measurement variability when compared to the EZ area and OS volume measurements. For the dice similarity analysis, the EZ band in each B-scan was marked from the OS layer segmentation to obtain a 2D EZ band annotation map [7] for the pixel-wise comparison between the different segmentation methods (DLM-MC, DLM only, and MG).

The EZ area and OS volume measurements obtained from DLM-MC by two human graders were then averaged and used to compare with that obtained from MG by two graders to assess the agreement between DLM-MC and MG. To compare the performance of the DLM only to that of MG, since each model was trained three times separately on the same datasets, three measurements of EZ area and OS volume were obtained by each DLM for an OCT volume scan, and the average of these three measurements was used to compare with that of MG.

The software package used for DLM training and validation, as well as for obtaining automatic segmentation and OS metrics measurements, is available at https://github.com/yzwang23/RFSW_RP_DLM/ (accessed on 26 October 2023) [31].

### 2.6. Data Analysis

The performance of DLM-MC as well as the DLM only in measuring the EZ area and OS volume from the test dataset was evaluated by comparing their measurement results to those of MG. Sørensen–Dice similarity analysis was employed to assess the agreement of the 2D EZ band annotation maps. Bland–Altman analysis, Pearson correlation, and linear regression were carried out to assess the agreement of the EZ area and OS volume measurements between the different methods.

## 3. Results

### 3.1. Dice Scores between EZ Band Segmentations by DLM-MC and by MG

**Figure 2:**
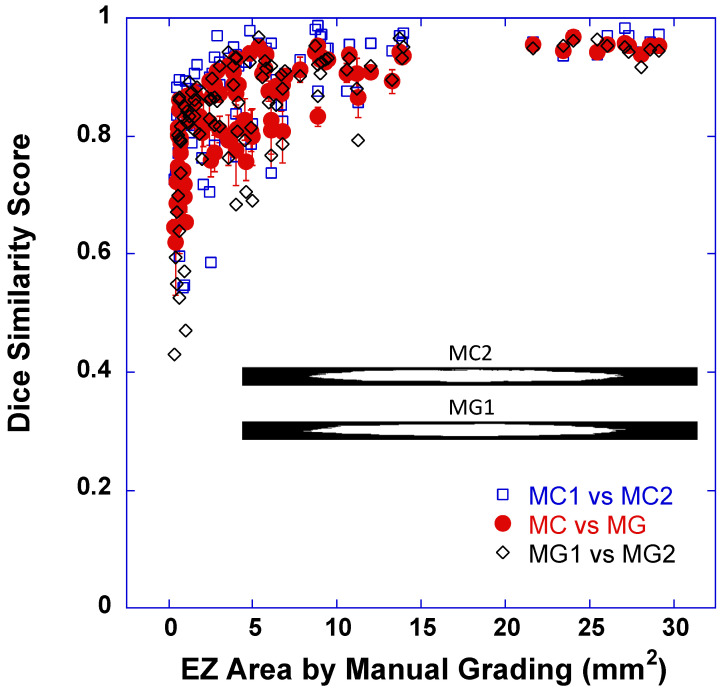
Dice similarity score for EZ band segmentation as a function of average EZ areas determined by conventional manual grading (MG) of two human graders. Closed red circles were the mean dice score between the EZ band area determined by DLM segmentation with manual correction (DLM-MC) and that by MG, and error bars indicate ±1 SD of the mean (average of 4 dice scores between two DLM-MCs and two MGs). Open blue squares were the dice scores between the EZ band areas determined by DLM-MC of grader 1 and that of grader 2, while open black diamonds were the dice scores between the EZ band areas determined by MG of grader 1 and that of grader 2. The inset images showed two examples of EZ band annotation maps (31 × 768 pixels). The top was from DLM-MC of the second grader (MC2) while the bottom was from MG of the first grader (MG1). The dice score was 0.9613 for this example.

The agreement between DLM-MC and MG in measuring the EZ areas was first evaluated with the dice similarity coefficient or dice score. The dice score was computed between the EZ band annotation maps (31 × 768, two examples are shown as inset images in Figure 2) determined by the individual DLM-MC and that determined by the individual MG for the 31 B-scan lines in each volume scan. Figure 2 plots the dice score as a function of the average EZ areas determined by the MG of two human graders. The closed red circles were the mean dice score between the EZ band annotation maps determined by DLM-MC and that determined by MG, and the error bars indicate ± 1 SD of the mean (average of four dice scores between two DLM-MCs and two MGs). Also plotted in Figure 2 are the dice scores between the EZ band annotation maps determined by two DLM-MCs, i.e., the DLM-MC of grader 1 and that of grader 2 (open blue squares), as well as the dice scores between the EZ band annotation maps determined by two MGs (open black diamonds).

As illustrated in Figure 2, when the EZ area was very small, the dice score varied significantly. The smaller the EZ, the smaller the dice coefficient. With the increase in the EZ area, the dice coefficient increased and gradually reached a plateau. The overall mean dice score (SD) for all the cases between DLM-MC and MG was 0.8524 (0.0821), which was comparable to the mean dice score (SD) of 0.8417 (0.1111) between the manual grading of two human graders (MG1 vs. MG2). When comparing DLM-MC1 and DLM-MC2, the mean dice score (D) was 0.8732 (0.0969). We also divided the EZ areas into two sub-groups using 1 mm^2^ as the criterion for easy comparison with the previously published results [7]. For the EZ area ≤ 1 mm^2^, the mean dice score (SD) between DLM-MC and MG was 0.7478 (0.0651). For the EZ area > 1 mm^2^, the mean dice score (SD) between DLM-MC and MG was 0.8799 (0.0614). Additional dice scores between MG1 and MG2, as well as between DLM-MC1 and DLM-MC2, can be found in Table 2, where the median dice score and the 25% and 75% quartiles are also reported.

### 3.2. Bland–Altman Plots—Limit of Agreement between DLM-MC and MG

**Figure 3:**
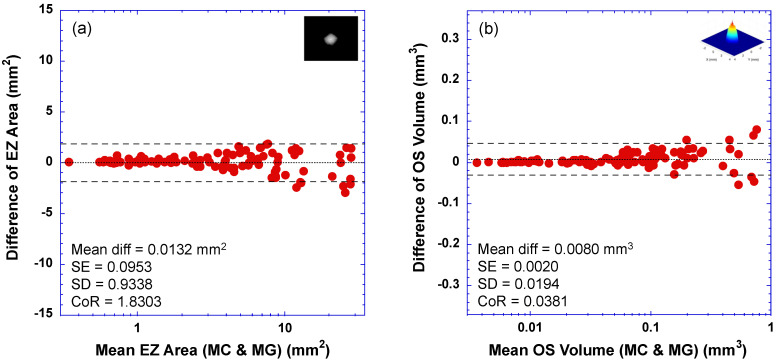
Bland–Altman plots of difference in measurements between DLM segmentation with manual correction (DLM-MC) and conventional manual grading (MG) versus their mean. (**a**) EZ area; (**b**) OS volume. The text insert in the plot lists the values of mean difference (Mean diff), standard error of the mean difference (SE), standard deviation of the mean difference (SD), and coefficient of repeatability (CoR, defined as 1.96 times the standard deviation of the difference). Dotted horizontal lines indicate the mean difference, and dashed horizontal lines represent ±95% limit of agreement (mean ± CoR).

The agreement between DLM-MC and MG in determining the EZ area and OS volume was also assessed using Bland–Altman plots. Figure 3 shows the Bland–Altman plots comparing the EZ area (2a) or the OS volume (2b) measured by DLM-MC to that measured by MG. In each plot, the horizontal axis is the mean EZ areas (2a) or the mean OS volumes (2b) measured by DLM-MC and MG, while the vertical axis is the difference in the EZ areas (2a) or OS volumes (2b) between DLM-MC and MG. The text in each plot lists the values of the mean difference (Mean diff), standard error of the mean difference (SE), standard deviation of the mean difference (SD), and coefficient of repeatability (CoR, defined as 1.96 times the standard deviation of the difference). The dotted horizontal lines indicate the mean difference, and the dashed horizontal lines represent the ±95% limit of agreement (mean ± CoR). For easy visualization of the data points of the smaller EZ areas, the horizontal axes of the plots in Figure 3 are in log scale.

DLM-MC showed an excellent agreement with MG for the EZ area measurement. The small mean difference between DLM-MC and MG (0.0132 mm^2^) was trivial because the mean difference ± 1.96 × SE included zero. For the OS volume measurement, DLM-MC showed a slight bias when compared to MG. When the CoR was normalized to the range of measurement (i.e., the range of vertical axes in Figure 3 is equal to the measurement range for the EZ area or OS volume), the relative CoR for the OS volume was smaller than that for the EZ area, as shown in Figure 3 with a narrower ±95% limit of agreement (dashed lines) for the OS volume, suggesting a tighter agreement between DLM-MC and MG for the OS volume measurement than for the EZ area measurement.

Table 3 and Table 4 list the summary results of the Bland–Altman analysis, including both the CoR and the mean difference (SD), as well as the mean absolute error (SD) for the EZ area and OS volume measurements, respectively. The mean absolute differences between DLM-MC and MG for the EZ area and OS volume measurements were in close agreement with that between the two MGs, as shown in Table 3 and Table 4.

### 3.3. Correlation and Linear Regression between DLM-MC and MG

The agreement between DLM-MC and MG in the measurement of the EZ area and OS volume was further evaluated with Pearson correlation analysis and linear regression. Table 3 and Table 4 also summarize the results of the correlation coefficients and linear regression slopes as well as their 95% confidence intervals (95% CI) for the EZ area and OS volume measurements, respectively. The coefficients of determination (*R*^2^) are also listed in Table 3 and Table 4.

In general, the results in Table 3 and Table 4 show that the EZ area and OS volume determined by DLM-MC was highly correlated with that determined by MG (r > 0.99). The linear regression slope was 0.96 for the EZ area measurements and close to 1 for the OS volume (95% CI included 1). The coefficients of determination *R*^2^ were larger than 0.98, suggesting that the agreement between DLM-MC and MG was 98% or higher for both the EZ area and the OS volume measurements.

### 3.4. DLM Only vs. MG

**Figure 4:**
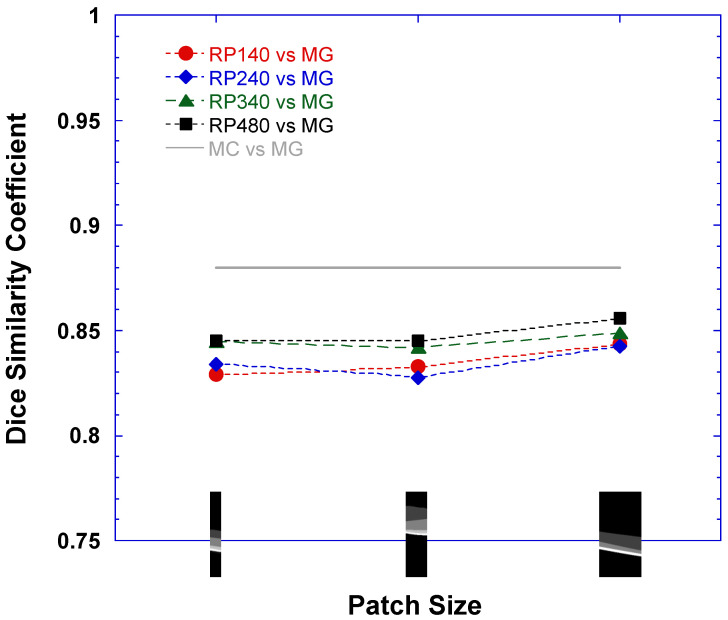
Dice similarity score for various DLMs vs. manual grading (MG) of two human graders for EZ area > 1 mm^2^. Horizontal axis lists three patch sizes, 256 × 32, 256 × 64, and 256 × 128, used in U-Net training in each of four RP models: RP140 (closed red circles), RP240 (closed blue diamonds), RP340 (closed green triangles), and RP480 (closed black squares). As a reference, solid gray line indicates the dice score between the EZ band measurements by DLM segmentation with manual correction (DLM-MC) and by MG.

The same methods used in Section 3.1, Section 3.2 and Section 3.3 were employed to assess the agreement between the DLM only and MG in the determination of the EZ area and OS volume. In addition to the dice similarity scores for comparing DLM-MC and MG, Table 2 also lists the detailed dice scores of the EZ band annotation maps between the various DLMs tested in this study and MG. Figure 4 plots the dice scores for four RP models vs. MG for the EZ area > 1 mm^2^. The horizontal axis in Figure 4 is the size of the image patches used in training the U-Net in each of the four models, i.e., 256 × 32, 256 × 64, and 256 × 128 from left to right. For comparison, the solid gray line in Figure 4 indicates the dice score of the EZ band measurements between DLM-MC and MG.

It is evident from Table 2 and Figure 4, with the increase in the training dataset size, that the performance of the DLMs improved when compared to MG. In addition, the DLMs trained with 256 × 128 image patches showed an improvement of the dice scores. However, the DLM only in this study still did not reach the performance level of DLM-MC when compared to MG.

**Figure 5:**
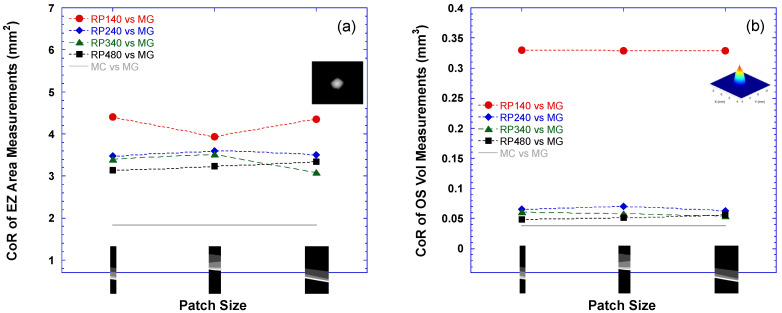
Coefficient of repeatability (CoR) of EZ area (**a**) and OS volume (**b**) measurements by various DLMs vs. manual grading (MG). Horizontal axis lists three patch sizes, 256 × 32, 256 × 64, and 256 × 128, used in U-Net training in each of four RP models: RP140 (closed red circles), RP240 (closed blue diamonds), RP340 (closed green triangles), and RP480 (closed black squares). As a reference, solid gray lines indicate CoR between the EZ area (**a**) or OS volume (**b**) measurements by DLM segmentation with manual correction (DLM-MC) and by MG.

Figure 5 plots the coefficient of repeatability (CoR) from the Bland–Altman analysis, which compared the performances of the various DLMs to MG in measuring the EZ area (4a) and OS volume (4b). As a reference, the solid gray lines in Figure 5 indicate the CoR between DLM-MC and MG. As with the findings for the dice scores, the performance of the DLM improved with the increase in the size of the training datasets. Figure 5 shows that the CoR decreased with the increase in the training dataset size, indicating closer agreement. For the EZ area, the improvement was relatively steady, while for the OS volume there was an initial big improvement step when the training dataset changed from RP140 to RP240; then, the improvement slowed down. On the other hand, the changing image patch size had a minimal impact on the CoR for the various DLMs. The average CoRs for the EZ area measurements across the three different patch sizes were 4.192, 3.526, 3.330, and 3.235 for RP140, RP240, RP340, and RP480, respectively. The average CoRs for the OS volume measurements across the three different patch sizes were 0.329, 0.066, 0.058, and 0.052 for RP140, RP240, RP340, and RP480, respectively.

In addition to the CoR, Table 3 and Table 4 include the summary results of the mean difference (SD), mean absolute error (SD), correlation coefficients, and linear regression slopes for the EZ area and OS volume measurements, respectively. The results in these tables also reveal that with the increase in the training image dataset size, the correlation coefficient increased and the linear regression slope became steeper and closer to 1. However, despite the performance improvement with the increase in the training dataset size, the DLM still fell behind DLM-MC when compared to MG.

## 4. Discussion

The results of this study demonstrated that both the EZ area and the OS volume measurements generated from the manual correction of the deep learning model’s automatic segmentation were in excellent agreement with those of the conventional manual segmentation by the human graders. The mean dice similarity score between DLM-MC and MG for all the EZ area measurements was 0.8524, which was comparable to that between the two human graders (0.8417). The coefficient of repeatability (CoR) between DLM-MC and MG was slightly smaller than that between the two manual graders for the EZ area measurement (1.8303 vs. 2.1629) and was almost identical for the OS volume measurement (0.0381 vs. 0.0389). The additional analyses of the agreement also revealed that the correlation coefficient and linear regression slope between DLM-MC and MG were comparable to those between the two human graders. As we reported previously, the average time (SD) of the manual correction of the DLM segmentation of the EZ band and pRPE boundary line was 4.10 (2.04) minutes for a low-density (31-line) volume scan and 9.33 (1.76) minutes for a high-density (121-line) volume scan [31]. Based on the time needed for our DLM to segment a B-scan image on an iMac Pro computer [27], it would take an average of 0.79 and 4.70 min for the U-Net and the hybrid model to segment a high-density (121-line) volume scan, respectively. Combining DLM segmentation with manual correction will still take much less time than the conventional manual grading; it typically takes hours for a human grader to segment a high-density volume scan obtained from patients with RP, based on our experience. The time for DLM segmentation can be significantly reduced with a more powerful computing system and with GPU acceleration [37,38]. Hence, the manual correction of automatic segmentation by a deep learning model can potentially be a new segmentation method to obtain reliable EZ area and OS volume measurements in RP to reduce the burden of human graders.

The hybrid model employed in this study consists of two CNN models: a traditional U-Net [33] for initial, fast semantic segmentation and a sliding-window (SW) CNN [34] for refinement. Comparing the performance of the U-Net to that of the hybrid model revealed that these two models are comparable to each other in terms of the measuring of OS metrics. For instance, the mean difference (SD) between the RP480 U-Net and the RP480 hybrid model was 0.0073 (0.0096) mm^2^ for the EZ area measurement and 0.0002 (0.0003) mm^3^ for the OS volume measurement, with a correlation coefficient of 1.0 between the two models. The CoR between the two models was 0.0188 mm^2^ for the EZ area and 0.0005 mm^3^ for the OS volume. This finding is consistent with our previous discovery that the segmentation of inner retinal layer boundaries benefits much less from the SW model refinement when compared to the segmentation of the inner limiting membrane (ILM) [27], suggesting that manual correction of the EZ and pRPE could be performed on the U-Net segmentation rather than on the hybrid model segmentation to further save time.

The pattern of dice score changes with the increase in the EZ area, as shown in Figure 2, is similar to what we reported previously, where the EZ area determined by the RP340 model was compared to that by a reading center [7], except that very small dice scores (<0.4) are not present in Figure 2 due to manual correction. We proposed that such a change in dice score resembles the behavior predicted by a simple lateral shift model for a dice score with a fixed shift of 0.315 mm between two same-sized circles [7]. Our previous work also showed that the mean difference in EZ width measured by the DLM and by MG was around 0.2 to 0.3 mm [27,30]. As long as there is a positional difference in EZ band segmentation between two graders, a smaller dice score is expected for a smaller EZ size. With the increase in EZ size, the negative impact of the constant positional difference in EZ band segmentation between the two graders on EZ band similarity becomes smaller, resulting in a higher dice score. On the other hand, a fixed shift model also predicts the increase in the absolute difference in EZ area measurements with the increase in EZ area, as shown in the Bland–Altman plot of Figure 3a.

The results of this study also demonstrated that the increase in the training dataset size can lead to the performance improvement of the DLM for the EZ area and OS volume measurements when compared to manual grading. However, the improved performance of the DLM shown in this study with the increased training dataset was still not as good as either DLM-MC or MG. Furthermore, the performance improvement seemed to slow down with the further increase in the size of the training dataset. Hence, manual checking and correction of DLM segmentation may be still needed in order to obtain reliable measurements before the DLM alone can reach the performance level close to DLM-MC or MG. One of the limitations of the current study was that all the training images were generated from mid-line B-scans only, which may miss some features in OCT scans that are mainly presented in off-center B-scans. By adding off-center B-scans from volume scans to the training dataset, the further performance improvement of the DLM is possible. In addition, the test dataset in this study consisted of 9 mm 31-line low-density volume scans only. The coarse separation of neighboring B-scans may introduce larger measurement variability and impact agreement analyses, especially for the segmentation by the DLM only. Additional datasets with high-density volume scans may be needed to further evaluate the agreement between the DLM and MG.

There are other limitations to this study. The main component of the hybrid model employed in this study is based on the original U-Net [33]. Since the introduction of the baseline U-Net in 2015, many variants have been proposed for medical image segmentation [39], including recurrent U-Net (RU-Net) and recurrent residual U-Net (R2U-Net) [40], inception U-Net [41,42], attention U-Net [43], etc. A recent study comparing eight different U-Net variants for the retinal layer segmentation of OCT images obtained from healthy participants and patients with macular degeneration suggests that the baseline U-Net is an optimal choice for OCT retinal layer segmentation in practice, and the state-of-the-art models do not appear to provide a clear benefit for such an application [44]. Nevertheless, it is still worth making a comparison with a different U-Net variant as a component of the hybrid model for the segmentation of OCT scan images obtained from patients with RP. An improved U-Net could potentially improve the hybrid model’s performance, which in turn could reduce the time needed for manual correction. Furthermore, because the main purpose of this study was to assess the performance of the manual correction of DLM segmentation as well as the effect of training dataset size, less effort was put into evaluating the performance of the DLM model itself with other metrics, such as ablation studies [45] to understand the contribution of the component to the overall system and the robustness of our model. Further work is needed in this area.

## 5. Conclusions

In summary, the manual correction of the automatic segmentation of the deep learning model can generate EZ area and OS volume measurements comparable to those of conventional manual grading in RP. Because DLM-MC can significantly save time for retinal layer segmentation from OCT scan images, it has the potential to reduce the burden of human graders when obtaining quantitative measurements of biomarkers for assessing disease progression and treatment outcomes. DLM-MC can also facilitate the study of the structure and function relationship. With a further increase in the size of the training datasets by including off-center B-scan images, we anticipate an additional improvement of the model’s automatic segmentation capability, and the DLM may eventually generate acceptable EZ area and OS volume measurements without manual correction.

## Figures and Tables

**Table 1:** Training datasets with the details, including patient composition, B-scan composition, and number of image patches for training and validation. “#” in the table means “number of”.

Training Dataset	# Patients						# Mid-Line B-Scans			Patch Horizontal Shift & # Patches for U-Net Training						# Patches for SW Model Training
	Total	adRP	arRP	XLRP	Isolated	Normal	Total	High-Speed (768 A-Scans)	High-Res (1536 A-Scans)	Patch Size: 256 × 32		Patch Size: 256 × 64		Patch Size: 256 × 128		
										Overlap (pixels)	# Patches	Overlap (pixels)	# Patches	Overlap (pixels)	# Patches	
RP140	140	50	15	15	30	30	280	183	97	28	300,369	56	148,705	112	72,179	1,646,562
RP240	240	50	30	20	120	20	480	305	175	28	527,504	56	261,658	112	127,691	2,878,002
RP340	340	80	40	30	170	20	680	446	234	28	737,084	56	365,520	112	178,242	3,984,084
RP480	480	130	55	45	200	50	960	629	331	28	1,037,453	56	514,225	112	250,421	5,630,646

**Table 2:** Dice similarity score of EZ band segmentation between DLM-MC and MG, between two MGs, and between two MCs, as well as between DLM only and MG. DLM-MC: deep learning model segmentation with manual correction; MG: conventional manual grading.

Dice Score (EZ Band Segmentation)		All Cases		Cases with EZ Area ≤ 1 mm^2^		Cases with EZ Area > 1 mm^2^	
		Mean (SD)	Median (Q1, Q3)	Mean (SD)	Median (Q1, Q3)	Mean (SD)	Median (Q1, Q3)
MC vs. MG		0.8524 (0.0821)	0.8674 (0.8052, 0.9212)	0.7478 (0.0651)	0.7446 (0.6963, 0.7970)	0.8799 (0.0614)	0.8875 (0.8319, 0.9344)
MG1 vs. MG2		0.8417 (0.1111)	0.8642 (0.8014, 0.9201)	0.7210 (0.1284)	0.7905 (0.6199, 0.8054)	0.8735 (0.0810)	0.8928 (0.8332, 0.9306)
MC1 vs. MC2		0.8732 (0.0969)	0.8902 (0.8341, 0.9486)	0.7791 (0.1083)	0.8208 (0.7354, 0.8559)	0.8979 (0.0771)	0.9239 (0.8670, 0.9561)
RP140 vs. MG	256 × 32	0.7859 (0.1276)	0.8124 (0.7198, 0.8896)	0.6212 (0.0833)	0.6249 (0.5672, 0.6803)	0.8293 (0.0987)	0.8513 (0.7756, 0.9092)
	256 × 64	0.7854 (0.1383)	0.8174 (0.7185, 0.9006)	0.6044 (0.1148)	0.6074 (0.4722, 0.7006)	0.8330 (0.0992)	0.8609 (0.7721, 0.9158)
	256 × 128	0.7847 (0.1504)	0.8390 (0.7261, 0.9003)	0.5614 (0.1254)	0.5578 (0.4897, 0.6338)	0.8435 (0.0887)	0.8598 (0.8112, 0.9073)
RP240 vs. MG	256 × 32	0.8002 (0.1047)	0.8148 (0.7382, 0.8935)	0.6725 (0.0883)	0.6695 (0.6055, 0.7440)	0.8338 (0.0801)	0.8392 (0.7917, 0.9089)
	256 × 64	0.7915 (0.1122)	0.8051 (0.7310, 0.8847)	0.6544 (0.0935)	0.6711 (0.6042, 0.7210)	0.8276 (0.0859)	0.8328 (0.7893, 0.9064)
	256 × 128	0.8014 (0.1105)	0.8339 (0.7183, 0.8976)	0.6460 (0.0816)	0.6767 (0.5955, 0.6999)	0.8423 (0.0752)	0.8510 (0.8042, 0.8999)
RP340 vs. MG	256 × 32	0.8055 (0.1133)	0.8293 (0.7550, 0.8949)	0.6561 (0.0877)	0.6600 (0.5929, 0.7064)	0.8448 (0.0823)	0.8518 (0.8095, 0.9150)
	256 × 64	0.8012 (0.1168)	0.8280 (0.7339, 0.8997)	0.6461 (0.0932)	0.6629 (0.6221, 0.6954)	0.8420 (0.0835)	0.8506 (0.8106, 0.9072)
	256 × 128	0.8096 (0.1114)	0.8375 (0.7391, 0.9043)	0.6608 (0.0897)	0.6831 (0.6130, 0.7215)	0.8487 (0.0789)	0.8628 (0.8126, 0.9096)
RP480 vs. MG	256 × 32	0.8115 (0.1065)	0.8294 (0.7557, 0.8970)	0.6827 (0.0910)	0.6974 (0.6245, 0.7500)	0.8454 (0.0818)	0.8611 (0.7945, 0.9100)
	256 × 64	0.8027 (0.1208)	0.8336 (0.7455, 0.9005)	0.6411 (0.1094)	0.6568 (0.5704, 0.7371)	0.8452 (0.0815)	0.8559 (0.8058, 0.9101)
	256 × 128	0.8189 (0.1004)	0.8369 (0.7646, 0.9069)	0.6783 (0.0791)	0.6815 (0.6305, 0.7320)	0.8559 (0.0672)	0.8558 (0.8225, 0.9132)

**Table 3:** Summary results of the agreement analyses for EZ area measurement, including coefficients of repeatability (CoR), mean difference (SD), mean absolute error (SD), correlation coefficients (95% CI), coefficients of determination (*R*^2^), and linear regression slopes (95% CI). CI: confidence interval. SD: standard deviation.

Agreement of EZ Area Measurements		CoR (mm^2^)	Mean Difference (SD) (mm^2^)	Mean Absolute Error (SD) (mm^2^)	Correlation Coeffiicent *r* (95% CI)	*R* ^2^	Linear Regression Slope (95% CI)
MC vs. MG		1.8303	0.0132 (0.9338)	0.6561 (0.6612)	0.9928 (0.9892–0.9952)	0.9856	0.9598 (0.9399–0.9797)
MG1 vs. MG2		2.1629	−0.5320 (1.1035)	0.7958 (0.9294)	0.9913 (0.9869–0.9942)	0.9826	0.9344 (0.9131–0.9556)
MC1 vs. MC2		2.8368	0.0969 (1.4474)	1.1315 (0.9003)	0.9809 (0.9714–0.9872)	0.9621	0.9736 (0.9405–1.0067)
RP140 vs. MG	256 × 32	4.4089	−1.2443 (2.2494)	1.4211 (2.1409)	0.9662 (0.9497–0.9774)	0.9335	0.7939 (0.7576–0.8302
	256 × 64	3.9315	−0.9377 (2.0059)	1.3096 (1.7830)	0.9784 (0.9678–0.9856)	0.9573	0.7972 (0.7684–0.8251)
	256 × 128	4.2348	−1.3690 (2.1606)	1.5214 (2.0550)	0.9801 (0.9703–0.9867)	0.9606	0.7617 (0.7353–0.7881)
RP240 vs. MG	256 × 32	3.4719	−1.6201 (1.7714)	1.6400 (1.7529)	0.9820 (0.9730–0.9880)	0.9643	0.8310 (0.8035–0.8584)
	256 × 64	3.5993	−1.7114 (1.8364)	1.7513 (1.7979)	0.9799 (0.9700–0.9866)	0.9602	0.8272 (0.7983–0.8560)
	256 × 128	3.5077	−1.5654 (1.7896)	1.6040 (1.7548)	0.9817 (0.9700–0.9866)	0.9637	0.8283 (0.8008–0.8558)
RP340 vs. MG	256 × 32	3.3957	−1.5381 (1.7325)	1.5720 (1.7015)	0.9821 (0.9732–0.9880)	0.9645	0.8391 (0.8115–0.8667)
	256 × 64	3.5157	−1.6574 (1.7937)	1.6811 (1.7713)	0.9845 (0.9768–0.9897)	0.9692	0.8142 (0.7894–0.8391)
	256 × 128	3.0787	−1.5493 (1.5708)	1.5714 (1.5484)	0.9880 (0.9820–0.9920)	0.9761	0.8409 (0.8184–0.8634)
RP480 vs. MG	256 × 32	3.1337	−1.5201 (1.5988)	1.5625 (1.5570)	0.9843 (0.9765–0.9895)	0.9688	0.8569 (0.8306–0.8833)
	256 × 64	3.2281	−1.5422 (1.6470)	1.6031 (1.5872)	0.9826 (0.9740–0.9884)	0.9655	0.8563 (0.8286–0.8840)
	256 × 128	3.3433	−1.5492 (1.7058)	1.5916 (1.6659)	0.9844 (0.9767–0.9896)	0.9690	0.8324 (0.8069–0.8079)

**Table 4:** Summary results of the agreement analyses for OS volume measurement, including coefficients of repeatability (CoR), mean difference (SD), mean absolute error (SD), correlation coefficients (95% CI), coefficients of determination (*R*^2^), and linear regression slopes (95% CI). CI: confidence interval. SD: standard deviation.

Agreement of OS Volume Measurements		CoR (mm^3^)	Mean Difference (SD) (mm^3^)	Mean Absolute Error (SD) (mm^3^)	Correlation Coeffiicent *r* (95% CI)	*R* ^2^	Linear Regression Slope (95% CI)
MC vs. MG		0.0381	0.0080 (0.0194)	0.0137 (0.0160)	0.9938 (0.9906–0.9958)	0.9876	1.0104 (0.9909–1.0298)
MG1 vs. MG2		0.0389	0.0002 (0.0198)	0.0128 (0.0151)	0.9933 (0.9899–0.9955)	0.9866	0.9930 (0.9732–1.0129)
MC1 vs. MC2		0.0265	−0.0043 (0.0135)	0.0097 (0.0103)	0.9970 (0.9955–0.9980)	0.9940	0.9874 (0.9743–1.0005)
RP140 vs. MG	256 × 32	0.3300	−0.1013 (0.1683)	0.1058 (0.1656)	0.5822 (0.4321–0.7009)	0.3389	0.0140 (0.0106–0.0173)
	256 × 64	0.3288	−0.1027 (0.1677)	0.1056 (0.1659)	0.6200 (0.4791–0.7298)	0.3845	0.0177 (0.0138–0.0215)
	256 × 128	0.3290	−0.1014 (0.1679)	0.1049 (0.1656)	0.6168 (0.4751–0.7274)	0.3805	0.0169 (0.0132–0.0206)
RP240 vs. MG	256 × 32	0.0653	−0.0119 (0.0333)	0.0170 (0.0310)	0.9829 (0.9745–0.9886)	0.9661	0.9019 (0.8729–0.9308)
	256 × 64	0.0700	−0.0141 (0.0357)	0.0196 (0.0330)	0.9800 (0.9701–0.9866)	0.9603	0.8976 (0.8664–0.9289)
	256 × 128	0.0623	−0.0113 (0.0318)	0.0175 (0.0288)	0.9844 (0.8815–0.9372)	0.9690	0.9093 (0.8815–0.9372)
RP340 vs. MG	256 × 32	0.0608	−0.0070 (0.0310)	0.0162 (0.0273)	0.9836 (0.9755–0.9890)	0.9675	0.9447 (0.9151–0.9744)
	256 × 64	0.0581	−0.0097 (0.0296)	0.0157 (0.0269)	0.9863 (0.8933–0.9460)	0.9728	0.9197 (0.8933–0.9460)
	256 × 128	0.0541	−0.0090 (0.0276)	0.0152 (0.0247)	0.9880 (0.9038–0.9535)	0.9762	0.9286 (0.9038–0.9535)
RP480 vs. MG	256 × 32	0.0487	−0.0049 (0.0248)	0.0149 (0.0205)	0.9894 (0.9841–0.9929)	0.9788	0.9757 (0.9511–1.0003)
	256 × 64	0.0513	−0.0060 (0.0262)	0.0155 (0.0219)	0.9882 (0.9403–0.9915)	0.9766	0.9659 (0.9403–0.9915)
	256 × 128	0.0565	−0.0084 (0.0289)	0.0154 (0.0258)	0.9866 (0.9031–0.9558)	0.9734	0.9294 (0.9031–0.9558)

## Data Availability

The data presented in this study are available in the article or upon request.

## Abbreviations

2D	two-dimensional
3D	three-dimensional
ART	automatic real-time tracking
BM	Bruch’s membrane
CI	confidence interval
CNN	convolutional neural network
CoR	coefficient of repeatability
dINL	distal inner nuclear layer
DLM	deep learning model
DLM-MC	deep learning model segmentation with manual correction
EZ	photoreceptor ellipsoid zone
HVSL	Hood Visual Science Laboratory
ILM	inner limiting membrane
MC	manual correction
MG	conventional manual grading
OCT	optical coherence tomography
OS	photoreceptor outer segment
pRPE	proximal (apical) retinal pigment epithelium
R^2^	coefficients of determination
ReLU	rectified linear unit
RP	retinitis pigmentosa
RPE	retinal pigment epithelium
RPGR	Retinitis Pigmentosa GTPase Regulator
SD	standard deviation
SE	standard error
SW	sliding-window
XLRP	x-linked retinitis pigmentosa

## References

[1] Aleman T.S., Cideciyan A.V., Sumaroka A., Windsor E.A.M., Herrera W., White D.A., Kaushal S., Naidu A., Roman A.J., Schwartz S.B. (2008). Retinal Laminar Architecture in Human Retinitis Pigmentosa Caused by Rhodopsin Gene Mutations. Investig. Opthalmol. Vis. Sci..

[2] Jacobson S.G., Aleman T.S., Sumaroka A., Cideciyan A.V., Roman A.J., Windsor E.A.M., Schwartz S.B., Rehm H.L., Kimberling W.J. (2009). Disease Boundaries in the Retina of Patients with Usher Syndrome Caused by MYO7A Gene Mutations. Investig. Ophthalmol. Vis. Sci..

[3] Witkin A.J., Ko T.H., Fujimoto J.G., Chan A., Drexler W., Schuman J.S., Reichel E., Duker J.S. (2006). Ultra-high Resolution Optical Coherence Tomography Assessment of Photoreceptors in Retinitis Pigmentosa and Related Diseases. Arch. Ophthalmol..

[4] Birch D.G., Locke K.G., Wen Y., Locke K.I., Hoffman D.R., Hood D.C. (2013). Spectral-Domain Optical Coherence Tomography Measures of Outer Segment Layer Progression in Patients with X-Linked Retinitis Pigmentosa. JAMA Ophthalmol..

[5] Smith T.B., Parker M.A., Steinkamp P.N., Romo A., Erker L.R., Lujan B.J., Smith N. (2019). Reliability of Spectral-Domain OCT Ellipsoid Zone Area and Shape Measurements in Retinitis Pigmentosa. Transl. Vis. Sci. Technol..

[6] Menghini M., Jolly J.K., Nanda A., Wood L., Cehajic-Kapetanovic J., MacLaren R.E. (2020). Early Cone Photoreceptor Outer Segment Length Shortening in RPGR X-Linked Retinitis Pigmentosa. Ophthalmology.

[7] Wang Y.-Z., Birch D.G. (2022). Performance of Deep Learning Models in Automatic Measurement of Ellipsoid Zone Area on Baseline Optical Coherence Tomography (OCT) Images From the Rate of Progression of USH2A-Related Retinal Degeneration (RUSH2A) Study. Front. Med..

[8] Varela M.D., Sen S., De Guimaraes T.A.C., Kabiri N., Pontikos N., Balaskas K., Michaelides M. (2023). Artificial intelligence in retinal disease: Clinical application, challenges, and future directions. Graefe’s Arch. Clin. Exp. Ophthalmol..

[9] Ting D.S.W., Pasquale L.R., Peng L., Campbell J.P., Lee A.Y., Raman R., Tan G.S.W., Schmetterer L., Keane P.A., Wong T.Y. (2019). Artificial intelligence and deep learning in ophthalmology. Br. J. Ophthalmol..

[10] Gargeya R., Leng T. (2017). Automated Identification of Diabetic Retinopathy Using Deep Learning. Ophthalmology.

[11] Gulshan V., Peng L., Coram M., Stumpe M.C., Wu D., Narayanaswamy A., Venugopalan S., Widner K., Madams T., Cuadros J. (2016). Development and Validation of a Deep Learning Algorithm for Detection of Diabetic Retinopathy in Retinal Fundus Photographs. JAMA.

[12] Lee C.S., Baughman D.M., Lee A.Y. (2017). Deep learning is effective for the classification of OCT images of normal versus Age-related Macular Degeneration. Ophthalmol. Retina.

[13] De Fauw J., Ledsam J.R., Romera-Paredes B., Nikolov S., Tomasev N., Blackwell S., Askham H., Glorot X., O’Donoghue B., Visentin D. (2018). Clinically applicable deep learning for diagnosis and referral in retinal disease. Nat. Med..

[14] Christopher M., Bowd C., Belghith A., Goldbaum M.H., Weinreb R.N., Fazio M.A., Girkin C.A., Liebmann J.M., Zangwill L.M. (2020). Deep Learning Approaches Predict Glaucomatous Visual Field Damage from OCT Optic Nerve Head En Face Images and Retinal Nerve Fiber Layer Thickness Maps. Ophthalmology.

[15] Kawczynski M.G., Bengtsson T., Dai J., Hopkins J.J., Gao S.S., Willis J.R. (2020). Development of Deep Learning Models to Predict Best-Corrected Visual Acuity from Optical Coherence Tomography. Transl. Vis. Sci. Technol..

[16] Fang L., Cunefare D., Wang C., Guymer R.H., Li S., Farsiu S. (2017). Automatic segmentation of nine retinal layer boundaries in OCT images of non-exudative AMD patients using deep learning and graph search. Biomed. Opt. Express.

[17] Kugelman J., Alonso-Caneiro D., Read S.A., Hamwood J., Vincent S.J., Chen F.K., Collins M.J. (2019). Automatic choroidal segmentation in OCT images using supervised deep learning methods. Sci. Rep..

[18] Lee C.S., Tyring A.J., Deruyter N.P., Wu Y., Rokem A., Lee A.Y. (2017). Deep-learning based, automated segmentation of macular edema in optical coherence tomography. Biomed. Opt. Express.

[19] Schlegl T., Waldstein S.M., Bogunovic H., Endstrasser F., Sadeghipour A., Philip A.M., Podkowinski D., Gerendas B.S., Langs G., Schmidt-Erfurth U. (2018). Fully Automated Detection and Quantification of Macular Fluid in OCT Using Deep Learning. Ophthalmology.

[20] Masood S., Fang R., Li P., Li H., Sheng B., Mathavan A., Wang X., Yang P., Wu Q., Qin J. (2019). Automatic Choroid Layer Segmentation from Optical Coherence Tomography Images Using Deep Learning. Sci. Rep..

[21] Ngo L., Cha J., Han J.H. (2020). Deep Neural Network Regression for Automated Retinal Layer Segmentation in Optical Coherence Tomography Images. IEEE Trans. Image Process..

[22] Pekala M., Joshi N., Liu T.Y.A., Bressler N.M., DeBuc D.C., Burlina P. (2019). Deep learning based retinal OCT segmentation. Comput. Biol. Med..

[23] Shah A., Zhou L., Abramoff M.D., Wu X. (2018). Multiple surface segmentation using convolution neural nets: Application to retinal layer segmentation in OCT images. Biomed. Opt. Express.

[24] Viedma I.A., Alonso-Caneiro D., Read S.A., Collins M.J. (2022). Deep learning in retinal optical coherence tomography (OCT): A comprehensive survey. Neurocomputing.

[25] Camino A., Wang Z., Wang J., Pennesi M.E., Yang P., Huang D., Li D., Jia Y. (2018). Deep learning for the segmentation of preserved photoreceptors on en face optional coherence tomogrpahy in two inherited retinal diseases. Biomed. Opt. Express.

[26] Loo J.M., Jaffe G.J., Duncan J.L., Birch D.G., Farsiu S. (2022). Validation of a Deep Learning-Based Algorithm for Segmentation of The Ellipsoid Zone on Optical Coherence Tomography Images of an Ush2a-Related Retinal Degeneration Clinical Trial. Retina.

[27] Wang Y.-Z., Wu W., Birch D.G. (2021). A Hybrid Model Composed of Two Convolutional Neural Networks (CNNs) for Automatic Retinal Layer Segmentation of OCT Images in Retinitis Pigmentosa (RP). Transl. Vis. Sci. Technol..

[28] Wang Z., Camino A., Hagag A.M., Wang J., Weleber R.G., Yang P., Pennesi M.E., Huang D., Li D., Jia Y. (2018). Automated detection of preserved photoreceptor on optical coherence tomography in choroideremia based on machine learning. J. Biophotonics.

[29] Loo J., Fang L., Cunefare D., Jaffe G.J., Farsiu S. (2018). Deep longitudinal transfer learning-based automatic segmentation of photoreceptor ellipsoid zone defects on optical coherence tomography images of macular telangiectasia type 2. Biomed. Opt. Express.

[30] Wang Y.-Z., Galles D., Klein M., Locke K.G., Birch D.G. (2020). Application of a Deep Machine Learning Model for Automatic Measurement of EZ Width in SD-OCT Images of RP. Transl. Vis. Sci. Technol..

[31] Wang Y.-Z., Juroch K., Chen Y., Ying G.S., Birch D.G. (2023). Deep learning facilitated study of the rate of change in photoreceptor outer segment (OS) metrics in x-linked retinitis pigmentosa (xlRP). Investig. Ophthalmol. Vis. Sci..

[32] Hoffman D.R., Hughbanks-Wheaton D.K., Pearson N.S., Fish G.E., Spencer R., Takacs A., Klein M., Locke K.G., Birch D.G. (2014). Four-Year Placebo-Controlled Trial of Docosahexaenoic Acid in X-Linked Retinitis Pigmentosa (DHAX Trial). JAMA Ophthalmol..

[33] Ronneberger O., Fischer P., Brox T. (2015). U-Net: Convolutional Networks for Biomedical Image Segmentation. arXiv.

[34] Ciresan D.C., Gambardella L.M., Giusti A., Schmidhuber J., Pereira F., Burges C.J.C., Bottou L., Weinberger K.Q. (2012). Deep neural networks segment neuronal membranes in electron microscopy images. NIPS’12: Proceedings of the NIPS’12: 25th International Conference on Neural Information Processing System, Lake Tahoe, NV, USA, 3–6 December 2012.

[35] Ioffe S., Szegedy C. Batch normalization: Accelerating deep network training by reducing internal covariate shift. Proceedings of the 32nd International Conference on Machine Learning, Proceedings of Machine Learning Research.

[36] Yang Q., Reisman C.A., Chan K., Ramachandran R., Raza A., Hood D.C. (2011). Automated segmentation of outer retinal layers in macular OCT images of patients with retinitis pigmentosa. Biomed. Opt. Express.

[37] Chen Z., Wang J., He H., Huang X. A fast deep learning system using GPU. Proceedings of the 2014 IEEE International Symposium on Circuits and Systems (ISCAS).

[38] Pandey M., Fernandez M., Gentile F., Isayev O., Tropsha A., Stern A.C., Cherkasov A. (2022). The transformational role of GPU computing and deep learning in drug discovery. Nat. Mach. Intell..

[39] Siddique N., Paheding S., Elkin C.P., Devabhaktuni V. (2021). U-Net and Its Variants for Medical Image Segmentation: A Review of Theory and Applications. IEEE Access.

[40] Alom M.Z., Hasan M., Yakopcic C., Taha T.M., Asari V.K. (2018). Recurrent residual convolutional neural network based on u-net (r2u-net) for medical image segmentation. arXiv.

[41] Szegedy C., Liu W., Jia Y., Sermanet P., Reed S., Anguelov D., Erhan D., Vanhoucke V., Rabinovich A. Going deeper with convolutions. Proceedings of the IEEE Conference on Computer Vision and Pattern Recognition (CVPR).

[42] Zhao H., Sun N., Zhao Y., Kong X., Taubman D. (2017). Improved U-Net Model for Nerve Segmentation. Image and Graphics.

[43] Oktay O., Schlemper J., Folgoc L.L., Lee M., Heinrich M., Misawa K., Mori K., McDonagh S., Hammerla N.Y., Kainz B. (2018). Attention U-Net: Learning where to look for the pancreas. arXiv.

[44] Kugelman J., Allman J., Read S.A., Vincent S.J., Tong J., Kalloniatis M., Chen F.K., Collins M.J., Alonso-Caneiro D. (2022). A comparison of deep learning U-Net architectures for posterior segment OCT retinal layer segmentation. Sci. Rep..

[45] Meyes R., Lu M., de Puiseau C.W., Meise T. (2019). Ablation studies in artificial neural networks. arXiv.

